# Transcriptome Analysis Reveals Contrasting Plant Responses of *Sorghum bicolor* upon Colonization by Two *Formae Speciales* of *Sporisorium reilianum*

**DOI:** 10.3390/ijms23168864

**Published:** 2022-08-09

**Authors:** Alana Poloni, Ravindra Garde, Lukas Dorian Dittiger, Theresa Heidrich, Christian Müller, Frank Drechsler, Yulei Zhao, Tilottama Mazumdar, Jan Schirawski

**Affiliations:** 1 Department for Molecular Biology of Plant-Microbe Interaction, Albrecht-von-Haller Institute for Plant Sciences, Georg-August-University Göttingen, Julia-Lermontowa-Weg 3, 37077 Göttingen, Germany; 2 Department of Microbial Genetics, Institute of Applied Microbiology, RWTH Aachen University, Worringerweg 1, 52074 Aachen, Germany; 3 Department of Genetics, Matthias-Schleiden-Institute, Friedrich-Schiller-University Jena, Philosophenweg 12, 07743 Jena, Germany

**Keywords:** *Sporisorium reilianum*, sorghum, host specificity, transcriptome, real-time PCR, MapMan, GO term analysis, defense responses, endoplasmic reticulum, unfolded protein response

## Abstract

The biotrophic fungus *Sporisorium reilianum* exists in two host-adapted *formae speciales* that cause head smut in maize (*S. reilianum* f. sp. *zeae*; SRZ) and sorghum (*S. reilianum* f. sp. *reilianum*; SRS). In sorghum, the spread of SRZ is limited to the leaves. To understand the plant responses to each *forma specialis*, we determined the transcriptome of sorghum leaves inoculated either with SRS or SRZ. Fungal inoculation led to gene expression rather than suppression in sorghum. SRZ induced a much greater number of genes than SRS. Each *forma specialis* induced a distinct set of plant genes. The SRZ-induced genes were involved in plant defense mainly at the plasma membrane and were associated with the Molecular Function Gene Ontology terms chitin binding, abscisic acid binding, protein phosphatase inhibitor activity, terpene synthase activity, chitinase activity, transmembrane transporter activity and signaling receptor activity. Specifically, we found an upregulation of the genes involved in phospholipid degradation and sphingolipid biosynthesis, suggesting that the lipid content of the plant plasma membrane may contribute to preventing the systemic spread of SRZ. In contrast, the colonization of sorghum with SRS increased the expression of the genes involved in the detoxification of cellular oxidants and in the unfolded protein response at the endoplasmic reticulum, as well as of the genes modifying the cuticle wax and lipid composition through the generation of alkanes and phytosterols. These results identified plant compartments that may have a function in resistance against SRZ (plasma membrane) and susceptibility towards SRS (endoplasmic reticulum) that need more attention in the future.

## 1. Introduction

Sorghum is the fifth most produced cereal crop worldwide and is an extremely tolerant plant against drought and temperature stresses. The crop is utilized for human and animal nutrition, as well as for forage, non-food products and the generation of bioenergy [1]. About 62 million tons of sorghum grain are estimated to be produced in 2022/2023 (http://www.worldagriculturalproduction.com/crops/sorghum.aspx; accessed on 8 August 2022). This number would be even higher if part of the world’s harvest was not lost due to the attack by phytopathogens that decreased the amount and quality of the grains. Besides the already identified diseases, newly emerging pathogens will continue to appear, making the control of plant plagues a more difficult task.

One of the known pathogens of sorghum is *Sporisorium reilianum* ([Kühn] Langdon and Fullerton), a biotrophic smut fungus that causes head smut on sorghum (*Sorghum bicolor*), Sudan grass (*Sorghum sudanense*) and maize (*Zea mays*) [2]. The disease is characterized by the modification of inflorescences that, instead of producing seeds, harbor a sorus containing masses of brown teliospores, therefore destroying the entire harvest of the plant. The spores spread in the environment and can survive in the soil for several years [3]. Under favorable conditions, the diploid spores germinate and undergo meiosis to yield haploid yeast-like sporidia of different mating type that multiply by budding. Two yeast-like cells of different mating type can recognize each other by a pheromone–pheromone receptor system encoded on the a-mating type locus [4]. Mating partner recognition leads to the termination of budding growth and the formation of conjugation tubes that grow towards each other and fuse at their tips [4]. If the fused cells also differ in their b-mating type loci, they form a dikaryotic filament that constitutes the infectious agent of the fungus [4]. The fungus penetrates the plant and proliferates until it reaches the apical meristem without causing obvious symptoms [5]. Fungal proliferation leads to the formation of sori in the inflorescences, in which diploid fungal spores form after karyogamy. Interestingly, *S. reilianum* is found in two *formae speciales* with distinct host preferences [2,6,7]. *S. reilianum* f. sp. *reilianum* (SRS) can be isolated from sorghum and is able to sporulate in sorghum but does not generally do so in maize [7]. *S. reilianum* f. sp. *zeae* (SRZ) can be isolated from infected maize and is unable to produce spores in sorghum [6]. Sorghum plants respond to an infection with SRZ by the accumulation of phytoalexins in the infected leaves that is visible as reddish-brown dots near the infection site [7].

During the attempt to infect the plant, microbial phytopathogens are confronted with diverse defense responses that are induced by the host. These responses include stomatal closure, changes in ion fluxes across the plasma membrane, the activation of mitogen-activated protein kinases (MAPKs), an oxidative burst in the form of reactive oxygen species (ROS), the reinforcement of the cell wall through the deposition of callose and lignin, the induction of plant hormones, an expression of transcription factors (TF) and pathogenesis-related proteins (PR) and the production of antimicrobial compounds such as phytoalexins [8,9,10,11,12,13,14]. To successfully infect the host, the pathogen must be able to avoid or inactivate the plant defense reactions. A recent study showed that the host specificity of SRZ and SRS on maize and sorghum is achieved by different mechanisms and indicated that SRZ induces different physiologic defense responses in sorghum that are not induced by SRS [5].

To understand plant responses to invading pathogens, the study of gene expression is a valuable tool. In sorghum, a microarray analysis was used to study the effects of different stresses, such as drought [15], high salinity, osmotic stress, abscisic acid (ABA) [16], salicylic acid, methyl jasmonate, a precursor of ethylene [17] and a phloem-feeding green bug aphid [18,19]. Recently, the sequencing of RNA (RNAseq) appeared as a new and efficient approach for transcriptomic analysis in sorghum, since the plant genome has been sequenced and annotated [20,21]. RNAseq has been successfully employed in *S. bicolor* to study the effects of osmotic stress and ABA [22], cold tolerance [23] and the differences in nitrogen tolerance in different sorghum genotypes [24]. A transcriptome analysis also revealed a restricted expression of the genes involved in photorespiration in the bundle sheath cells of sorghum [25] and was used to evaluate the expression of senescence-associated genes [26]. Additionally, RNAseq was used to investigate the interaction of sorghum and the pathogenic fungus *Bipolaris sorghicola*, revealing the induction of several plant defense genes [27,28]. Furthermore, transcriptome analyses investigating alternative splicing [29], drought tolerance [30] and phosphorus starvation tolerance [31] are available.

To find out which processes are activated in sorghum when challenged with two *formae speciales* of *S. reilianum*, one that induces spore formation (SRS) and the other one that does not (SRZ), we performed a total RNAseq of SRS- or SRZ-inoculated leaves of *S. bicolor* and analyzed the data for differentially regulated plant genes. The analysis revealed entirely distinct gene expression scenarios in sorghum, where the interaction with the non-virulent *forma specialis* (SRZ) induced a multitude of molecular defense responses, hinting among others at the reorganization of the plasma membrane through exchanging phospholipids by sphingolipids, while the interaction with the virulent one (SRS) mainly induced genes involved in the detoxification of oxidative damage as well as in the unfolded protein response in the endoplasmic reticulum of the plant.

## 2. Results

### 2.1. Illumina Sequencing of Sorghum Leaves Colonized by Two Different Formae Speciales of S. reilianum

To understand the plant response to a *S. reilianum* infection, we investigated the transcriptional response of sorghum (*S. bicolor*) leaves by an RNAseq of RNA isolated at 3 days after inoculation with SRS, SRZ or water as the control. For each replicate, pieces of about 3 cm were collected from the inoculated leaves (Figure S1) of eight plants, frozen in liquid nitrogen and ground to a fine powder that was used to isolate the total RNA. Equal RNA amounts of three biological replicates were pooled prior to Illumina Next-Generation sequencing (GATC Biotech, Konstanz, Germany). After quality control, more than 44, 46 and 88 million reads were obtained, respectively, for the samples inoculated with SRS, SRZ and water. The obtained reads were mapped using the Tuxedo protocol [32] against the reference genome of *S. bicolor* Sorghum_bicolor_NCBIv3 (GCA_000003195.3) [21,33], consisting of 33,134 genes. Of all the designated sorghum genes, the expression of 29,663 (89%) was detected in one, two or all three conditions. More than 93% of all the obtained reads could be mapped to the reference genome sequence. In total, 39, 41 and 81 million unique reads could be mapped (Table 1).

**Figure 1:**
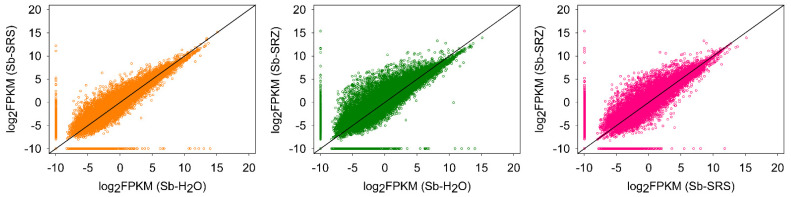
Comparison of FPKM of all sorghum genes at 3 days after inoculation. FPKM (fragments per kilobase of exon model per million mapped reads) values for transcripts detected in the comparison of Sb-SRS vs. Sb-H_2_O (**left**), Sb-SRZ vs. Sb-H_2_O (**middle**) or Sb-SRS vs. Sb-SRZ (**right**). A constant of 0.00001 was added to each FPKM to avoid zero scores in log calculations.

We compared the RPKM values obtained in the three conditions in a pairwise manner. More genes were regulated in the *S. reilianum* (SRZ- or SRS)-infected sample versus the water-inoculated control, and more in the SRZ-infected sample versus the SRS-infected sorghum (Figure 1).

### 2.2. Identification of Differentially Expressed Genes in Sorghum

To identify the genes specifically induced or repressed by each strain, we compared the *S. reilianum*-inoculated samples against the water-inoculated samples (Sb-SRS vs. Sb-H_2_O and Sb-SRZ vs. Sb-H_2_O) and the SRZ-infected samples against the SRS-infected samples (Sb-SRS vs. Sb-SRZ) using the Cuffdiff program. Differentially expressed genes with log2 fold change values > 1 or <−1 were considered as regulated. The Cuffdiff program uses the stringent Benjamini–Hochberg procedure to reduce the false discovery rate. Using these calculations, only 75, 450 and 297 genes, respectively, were detected as being significantly regulated in the comparisons of Sb-SRS vs. Sb-H_2_O, Sb-SRZ vs. Sb-H_2_O and Sb-SRS vs. Sb-SRZ.

**Figure 2:**
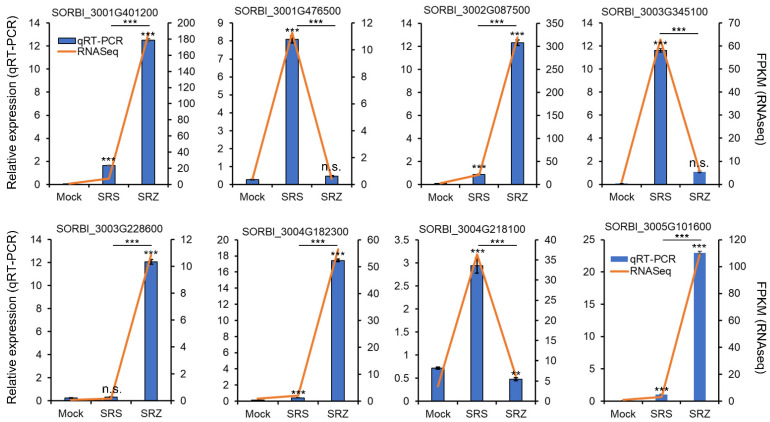
Validation of eight differentially regulated genes by qRT-PCR. Gene expression was measured by qRT-PCR after independent isolation of RNA of sorghum plants inoculated with water (mock), a combination of the mating compatible *S. reilianum* f. sp. *zeae* strains SRZ1_5-2 and SRZ2_5-1 (SRZ) or a combination of the mating compatible *S. reilianum* f. sp. *reilianum* strains SRS1_H2-8 and SRS2_H2-7 (SRS) relative to elongation factor 4 alpha (*ElF4α*; SORBI_3004G039400) and protein phosphatase 2 (*PP2A*; SORBI_3004G092500) and is given as relative normalized expression values (blue bars, left Y-axis). Error bars represent SEM of three independent biological replicates of three technical replicates each. Significance analysis was performed with a Student’s *t*-test relative to mock-inoculated samples, as indicated above by the error bars, and between SRS- and SRZ-infected samples, as indicated above by the respective bar graphs (n.s., non-significant; *, *p* < 0.05; **, *p* < 0.01; ***, *p* < 0.001). As comparison, the FPKM values of the RNAseq analysis are also indicated (orange line, right Y-axis).

To check whether these genes were reliably up- or downregulated, we repeated the sorghum infection experiment in a different greenhouse in three independent inoculation experiments, collected the leaf material of 17 plants for each inoculation and replicate and used it to isolate the total RNA. We selected eight putatively differentially regulated genes based on their difference in expression profile in the different samples analyzed by RNAseq and measured gene expression via qRT-PCR. The selected genes encoded a deformylating aldehyde oxygenase (SORBI_3004G218100), a BHLH domain-containing protein (SORBI_3001G476500), an uncharacterized protein (SORBI_3003G228600), a PR10a-ortholog (SORBI_3001G401200), an rRNA N-glycosidase (SORBI_3002G087500), a Fe^2+^-dependent oxoglutarate dioxygenase domain-containing protein (SORBI_3003G345100), an MLO-like protein (SORBI_3004G182300) and a dirigent protein (SORBI_3005G101600). According to the RNAseq evaluation, they should be upregulated only in Sb-SRZ (one gene; SORBI_3003G228600), more upregulated in Sb-SRZ than in Sb-SRS (four genes; SORBI_3001G401200, SORBI_3002G087500, SORBI_3004G182300 and SORBI_3005G101600), only upregulated in Sb-SRS (two genes; SORBI_3001G476500, SORBI_3003G345100) or most highly upregulated in Sb-SRS (one gene; SORBI_3004G218100). The selected genes varied in detected gene expression between 10 (SORBI_3003G228600) to over 300 FPKM (fragments of kilobase of transcripts per million mapped reads; SORBI_3002G087500). For all the selected genes, the gene expression profiles as independently determined by qRT-PCR corresponded well to the differences in gene expression as obtained in our pooled RNAseq experiment (Figure 2), confirming the reliability of the gene expression changes determined in the conducted RNAseq experiment.

**Figure 3:**
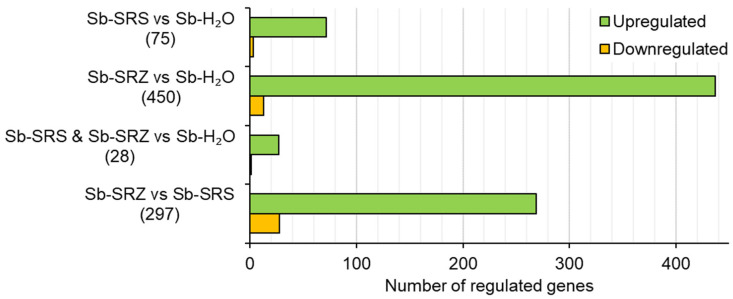
*S. reilianum* infection leads to gene expression in sorghum. Bar chart showing distribution of significantly differentially expressed (*p* ≤ 0.05) sorghum genes in the indicated comparisons. Infection with SRZ leads to more upregulated genes than infection with SRS.

In the comparison of Sb-SRS vs. Sb-H_2_O, 75 genes were differentially regulated. Of these, 72 (96%) were upregulated and 3 (4%) were downregulated (Figure 3). A similar ratio was found for SRZ vs. control, however, about six times as many genes were differentially regulated: of the 450 genes with differential regulation, 437 (97%) were upregulated and 13 (3%) downregulated (Figure 3). In total, 28 sorghum genes that were differentially regulated by inoculation with SRS were also regulated by inoculation with SRZ (Figure 3, Sb-SRS and Sb-SRZ vs. Sb-H_2_O). Of these 28 genes, 27 (96%) were upregulated and 1 (4%) downregulated in both samples relative to the mock-inoculated sorghum. No gene was upregulated in one sample and downregulated in the other.

We also compared the samples Sb-SRZ vs. Sb-SRS. In this comparison, 297 genes were differentially regulated, of which 269 (91%) were more strongly expressed in the SRZ-inoculated sorghum leaves while 28 (9%) were more strongly expressed in the SRS-inoculated sorghum leaves (Figure 3). In summary, the inoculation of sorghum with *S. reilianum* led to gene expression rather than gene suppression, and the inoculation with SRZ induced more gene expression changes than the inoculation with SRS.

### 2.3. GO-Term Analysis Reveals Fundamentally Different Strategies of Plant Response to Each Forma Specialis of S. reilianum

**Figure 4:**
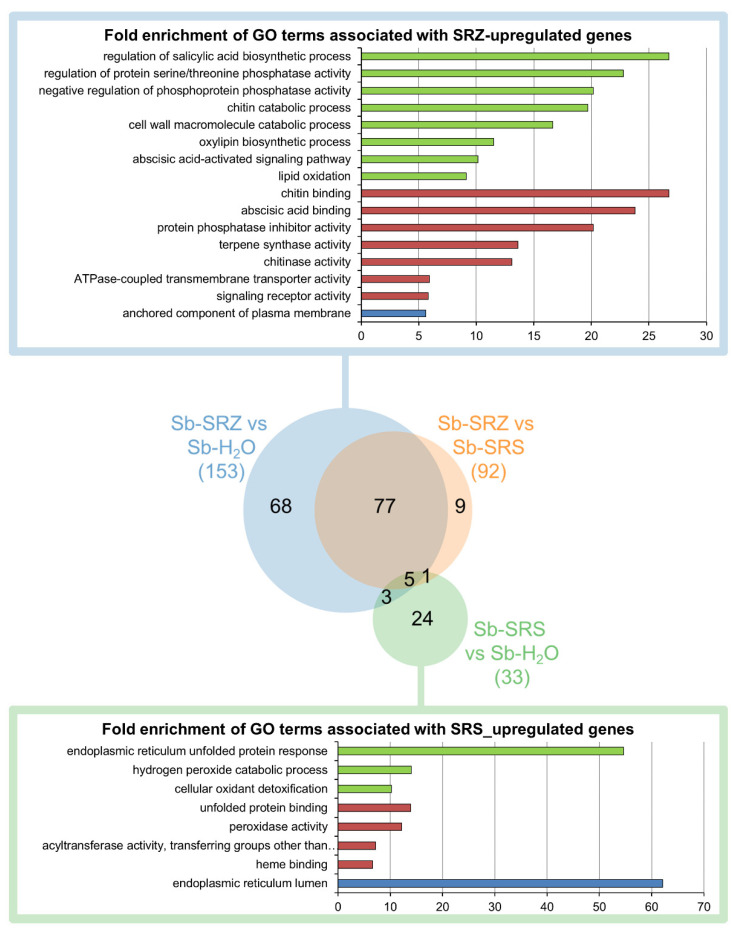
Overview of GO term analysis of differentially regulated sorghum genes. Fold enrichments of GO terms significantly overrepresented in the set of differentially regulated genes in all three GO categories (biological process, molecular function, cellular structure) for each of the three comparisons (Sb-SRZ vs. Sb-H_2_O, Sb-SRS-vs Sb-H_2_O, Sb-SRZ vs. Sb-SRS). Venn diagram created using the Venn Diagram Maker online from META-CHART. For the SRZ-induced genes, as well as for the SRS-induced genes, lists of the most significant GO terms are provided, selecting the youngest child term to represent a group of connected GO terms. Terms associated with biological process are represented in green, with molecular function in red and with cellular component in blue.

We analyzed the significantly regulated genes of the three conditions using the Gene Ontology Resource online. In the comparison of Sb-SRZ vs. Sb-H_2_O, more GO terms were significantly enriched among the upregulated genes than in any other comparison, whereas the comparison of Sb-SRS vs. Sb-H_2_O had the fewest significantly enriched GO terms (Figure 4). Most (68) GO terms appeared either in the comparison of Sb-SRZ vs. Sb-H_2_O or (77) appeared both in the comparison of Sb-SRZ vs. Sb-H_2_O and Sb-SRZ vs. Sb-SRS. This group of GO terms describes the function of genes that are more induced in sorghum by SRZ than by SRS. The most significant GO terms of this group in the biological process category were the “regulation of salicylic acid biosynthetic process”, “regulation of protein serine/threonine phosphatase activity”, “negative regulation of phosphoprotein phosphatase activity”, “chitin catabolic process”, “cell wall macromolecule catabolic process”, “oxylipin biosynthetic process”, “abscisic acid-activated signaling pathway” and “lipid oxidation”. In the molecular function category were terms that included “chitin binding”, “abscisic acid binding”, “protein phosphatase inhibitor activity”, “terpene synthase activity”, “chitinase activity”, “ATPase-coupled transmembrane transporter activity” and “signaling receptor activity”, whereas in the cellular component category the term “anchored component of plasma membrane” was the most prominent (Figure 4).

A small number of GO terms (9) appeared specifically in the comparison of Sb-SRZ vs. Sb-SRS and did not appear in the comparison of Sb-SRZ vs. Sb-H_2_O or Sb-SRS vs. Sb-H_2_O. These GO terms describe genes that are slightly downregulated by SRS and slightly upregulated by SRZ and included the term “2-alkenal reductase [NAD(P)+] activity” in the molecular function category. Of the 33 GO terms describing the genes that are upregulated by SRS, 24 were specific to the comparison of Sb-SRS vs. Sb-H_2_O. These included “endoplasmic reticulum unfolded protein response”, “hydrogen peroxide catabolic process” and “cellular oxidant detoxification” in the biological process category, as well as “unfolded protein binding”, “peroxidase activity” and “acyltransferase activity, transferring groups other than amino-acyl groups” in the molecular function category and “endoplasmic reticulum lumen” in the cellular component category (Figure 4).

This shows that each *forma specialis* of *S. reilianum* induced fundamentally different plant response strategies: whereas SRZ induced different defense reactions mainly at the plasma membrane, the sorghum infection by SRS led to oxidative damage and protein folding control in the endoplasmic reticulum of the plant.

### 2.4. MapMan Analysis of SRS- and SRZ-Upregulated Sorghum Genes

We then compared the sorghum genes that showed differential expression in any of the three conditions using the MapMan tool [34]. About half of the differentially expressed genes were either not annotated or were not assigned to any functional MapMan category. Only 32, 225 and 160 genes were recognized by MapMan and assigned to the functional categories of the comparisons of Sb-SRS vs. H_2_O, Sb-SRZ vs. H_2_O and Sb-SRZ vs. Sb-SRS, respectively.

**Figure 5:**
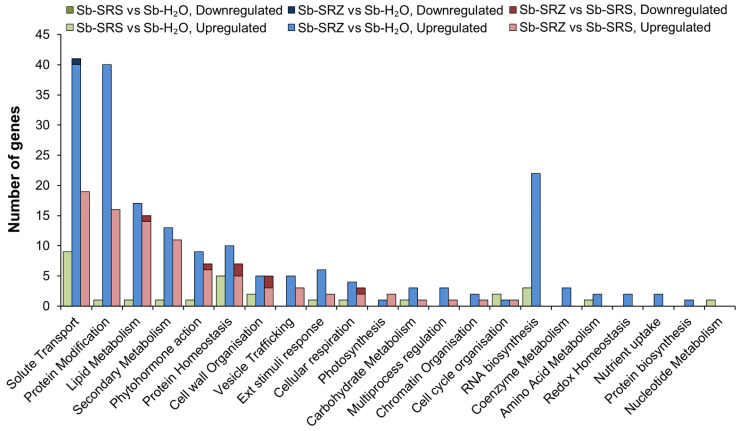
Bar chart showing number of differentially expressed sorghum genes classified in categories according to MapMan. Up- and downregulated genes of each comparison are presented in the same bar on top of each other. None of the downregulated genes of the comparison of Sb-SRS vs. Sb-H_2_O were assigned to any category.

Significantly regulated sorghum genes in the comparison of SRS versus mock-inoculated sorghum belonged to only 14 MapMan categories, while the sorghum infection with SRZ affected the genes of 21 MapMan categories, indicating that the SRZ infection affected a greater variety of processes. SRS induced most gene expression changes in the categories “solute transport”, “protein homeostasis” and “RNA biology”, while SRZ induced most gene expression changes in the categories “solute transport”, “protein modification”, “RNA biosynthesis”, “lipid metabolism”, “secondary metabolism”, “protein homeostasis” and “phytohormone action” (Figure 5). The category “solute transport” had the highest number of significantly upregulated genes in all three comparisons whereas the categories “protein modification”, “lipid metabolism”, “secondary metabolism” and “phytohormone action” contained mostly genes that were upregulated in the comparison of Sb-SRZ vs. Sb-SRS. Only a few (8) of the downregulated genes were associated with the MapMan categories. These mapped to the categories “protein homeostasis” (an FBX component of an SCF E3 ubiquitin ligase and an aminopeptidase of neutral/aromatic-hydroxyl amino acids) and “cell wall organization” (a xyloglucan endotransglucosylase/hydroxylase and a CER1 aldehyde decarboxylase component of the CER1-CER3 alkane forming complex), as well as “solute transport” (a metabolite transporter DTX), “lipid metabolism” (a 3-ketoacyl CoA Synthase), “phytohormone action” (Aux/IAA repressor component of the auxin receptor complex) and “cellular respiration” (a phosphoglycerate kinase) (Figure 5).

### 2.5. SRS Stimulates Glycolysis and Fatty Acid, Phytosterol and Cuticle Biosynthesis

**Figure 6:**
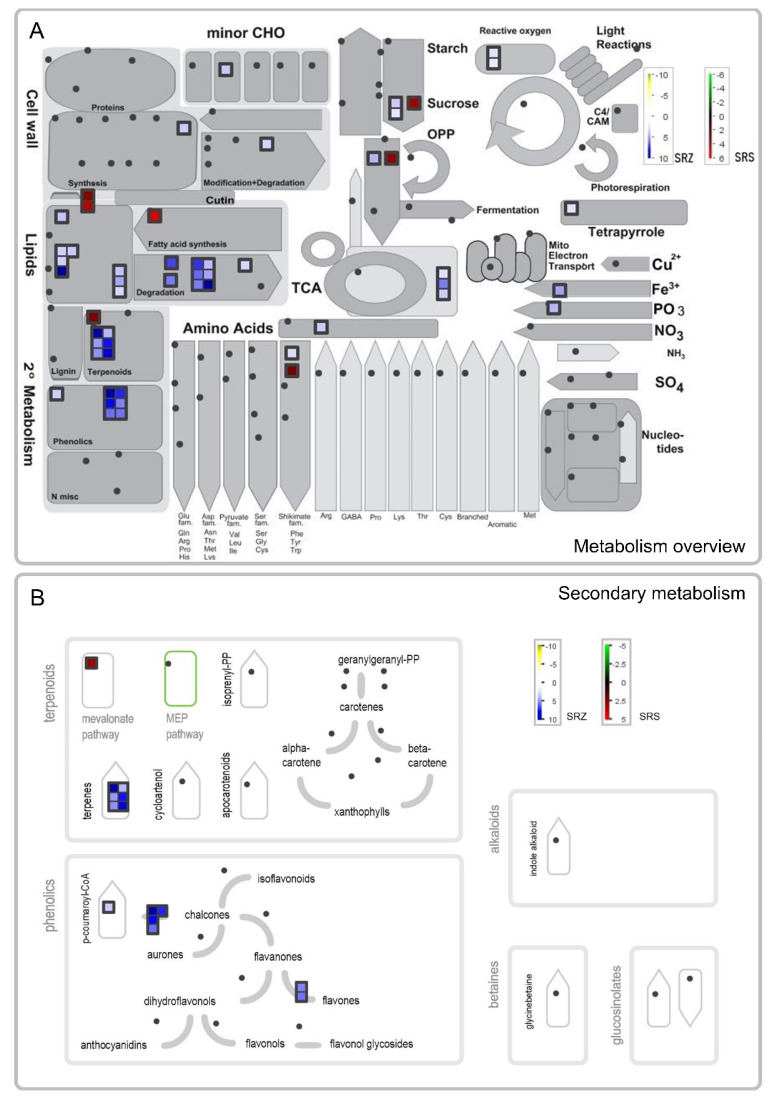
MapMan visualization of differentially expressed genes of *Sorghum bicolor* at 3 days after inoculation with *S. reilianum* f. sp. *reilianum* (SRS) or *S. reilianum* f. sp. *zeae* (SRZ). An overview of the metabolism is shown in (**A**), while (**B**) focuses on the genes involved in secondary metabolism. Significantly regulated genes are represented by squares whose color indicates the degree of regulation. In the comparison of Sb-SRS vs. Sb-H_2_O, the green–black–red color gradient indicates downregulated–not-regulated–upregulated genes, while in the comparison of Sb-SRZ vs. Sb-H_2_O, the used color gradient is yellow–white–blue. Dots indicate that the respective genes were not among the detected significantly regulated genes.

To better understand which pathways are affected by the two *formae speciales*, we visualized the changes in gene expression in the primary and secondary metabolism genes of the comparisons of Sb-SRZ vs. Sb-H_2_O and Sb-SRS vs. Sb-H_2_O. In the comparison of Sb-SRS vs. Sb-H_2_O, only seven upregulated genes were associated with primary or secondary metabolism in MapMan (Figure 6A). Of those seven genes, two were also upregulated in the comparison of Sb-SRZ vs. Sb-H_2_O, a 3-deoxy-D-arabino-heptulosonate 7-phosphate (DAHP) synthase putatively involved in wound healing [35] and an acid beta-fructofuranosidase, a cell wall invertase involved in the breakdown of sucrose [36] (Table 2). Five genes were upregulated only in the Sb-SRS vs. Sb-H_2_O comparison: CER1 and CER3, encoding an alkane-forming complex, a phosphoglycerate kinase involved in glycolysis, a 3-ketoacyl-CoA synthase involved in cuticle wax biosynthesis and a 3-hydroxy-3-methylglutaryl-CoA synthase, an enzyme putatively involved in plant phytosterol biosynthesis [37]. These genes are upregulated by the plant upon specific induction by SRS and therefore possibly constitute susceptibility genes that facilitate the entry or spread of the fungus [38].

### 2.6. SRZ Induces Membrane Reorganization through Exchange of Phospholipids by Cell-Death-Inducing Sphingolipids, as Well as Generation of Defense Terpenes and Phytoalexins

In the comparison of Sb-SRZ vs. Sb-H_2_O, we observed a strong upregulation of the genes involved in lipid metabolism and fatty acid degradation as well as the metabolism of terpenoids and phenolics that were specific to the SRZ-infected sample (Figure 6). Specifically, several phospholipases were upregulated that are involved in the degradation of phospholipids, as well as a monoacylglycerol lipase, a 3-ketoacyl-CoA thiolase and a caleosin lipid body surface protein that are also associated with lipid degradation (Table 2). In addition, three genes encoding fatty acid transporters and amino phospholipid ATPases (ALA) were upregulated that are involved in lipid trafficking (Table 2). In contrast, several enzymes for the biosynthesis of sphingolipids were upregulated, such as both subunits of the serine palmitoyltransferase, a phosphatidate phosphatase, a sphinganine C4-monooxygenase and an inositol phosphorylceramide synthase [39,40] (Table 2). This hints that SRZ induces major changes in the lipid composition of the plant that exchanges phospholipids by sphingolipids. Sphingolipids may play a critical role in defense against fungal pathogens by directing the cell to initiate programmed cell death [41].

Upregulated specifically in the comparison of Sb-SRZ vs. Sb-H_2_O were 13 genes associated with the secondary metabolism components terpenoids and phenolics (Figure 6A). Of the genes associated with terpenoids, all six genes were involved in the biosynthesis of terpenes and putatively encode mono-/sesquiterpene-/diterpene synthases (Figure 6B, Table 2). Of the seven genes associated with phenolics, one encodes a cinnamate 4-hydroxylase—a key enzyme in the synthesis of phytoalexins [42]—four encode chalcone synthases, putatively involved in the biosynthesis of flavonoid and isoflavonoid phytoalexins and of SA [43] and two encode type-I flavone synthases, suggested to mediate the cross talk between flavone and SA biosynthesis [44].

In addition, single genes involved in different processes appeared upregulated specifically in the comparison of Sb-SRZ vs. Sb-H_2_O (Table 2): a galactinol synthase, the key enzyme in the synthesis of oligosaccharides of the raffinose family that function as osmoprotectants in plant cells [45], a D-glucan synthase with a proposed function in cell wall biosynthesis and a pectin acetylesterase involved in cell wall modification, a PTH1 family phosphate transporter known to be expressed in sorghum leaves and suspected to play a role in phosphate mobilization in leaves (SbPT9) [46] and a glutamate decarboxylase, an enzyme involved in glutamate metabolism that has been identified to play a vital role in the plant–pathogen interaction [47]. In addition, several proteins that can be associated with ROS generation are upregulated specifically in SRZ-inoculated sorghum leaves: an H-type thioredoxin and a nucleoredoxin that might play a role in redox balance and the protection of antioxidant enzymes from ROS-induced oxidation, a chlorophyllase involved in the degradation of chlorophyll, a reaction putatively needed to protect the plant cells from an excess of H_2_O_2_ [48], a nicotianamine amino transferase, known to be involved in the siderophore biosynthesis and iron uptake of the plant [49], possibly compensating for the iron depletion within the plant cells due to the release of ferric iron to the apoplast to support H_2_O_2_ production [50] and three transcripts putatively encoding alternative oxidases known to have a role in diminishing the production of excessive H_2_O_2_ [51].

We observed a signature of the upregulation of jasmonic acid (JA) biosynthesis genes in the comparison of Sb-SRZ vs. Sb-H_2_O that was not seen in the comparison of Sb-SRS vs. Sb-H_2_O. Two genes encoding 13-lipoxygenase and allene oxide synthase that catalyze essential JA biosynthesis reactions in the chloroplasts were upregulated with fold changes of 2.6 and 5.5, respectively. In contrast, SA hydroxylase leading to decreased levels of SA was upregulated in the comparison of Sb-SRZ vs. Sb-H_2_O with a fold change value of 6.0. This shows that both fungal *formae speciales* are effectively downregulating SA and SA-induced defenses, normally mounted by plants against biotrophic pathogens. The increase in JA biosynthesis and SA degradation enzymes may also be an indirect response to cell death occurring due to local phytoalexin or sphingolipid accumulation.

To corroborate the putative induction of plant defenses by SRZ, we analyzed the datasets for changes in gene expression in membrane-spanning kinases. We observed that 22 membrane-spanning kinases were upregulated in the comparison of Sb-SRZ vs. Sb-H_2_O, of which 21 were not induced in the comparison of Sb-SRS vs. Sb-H_2_O. Only one membrane-spanning kinase was upregulated by both SRS and SRZ. The 22 membrane-spanning kinases that were significantly upregulated in the comparison of Sb-SRZ vs. Sb-H_2_O were grouped into 12 protein families (Table 3), known to be involved in the response to stress and/or pathogen defense, such as L-type lectin receptor kinases, MAP kinase kinase kinases, WAK-like kinases, S-domain kinases, PERK and CMCG kinase, DUF26 containing kinases, SNF1-related kinases and LRR kinases of groups VIII-1, Xc, XI and XII (Table 3). Of the intracellular kinases, nine were upregulated in the comparison of Sb-SRZ vs. Sb-H_2_O, and these were not detected in the comparison of Sb-SRS vs. Sb-H_2_O. These kinases belonged to the LRR, RLCK and DLSV groups, also known to be involved in pathogen and/or stress resistance (Table 3).

## 3. Discussion

Smut fungi have a limited host range, generally infecting one or only a few distinct plant species. This is also true for the two host-adapted *formae speciales* of *S. reilianum* that can cause disease in sorghum and some related wild grasses (*S. reilianum* f. sp. *reilianum*; SRS) or on maize (*S. reilianum* f. sp. *zeae*; SRZ) [2]. Both SRS and SRZ can penetrate the leaf surface of sorghum and multiply in the leaf tissue [5]. However, while SRS can spread in the plant towards the meristem, SRZ does not reach the meristematic tissue in sorghum. Instead, SRZ encounters defense responses of sorghum that include increased H_2_O_2_ formation, callose deposition and phytoalexin induction [5]. To better understand how sorghum reacts to the different strains of *S. reilianum* and how *S. reilianum* modulates plant gene expression, we performed a comparative transcriptome analysis of sorghum leaves colonized by SRS and SRZ.

We found a greater number of differentially expressed genes in the SRZ-infected samples as compared to those infected with SRS. A deeper analysis of the data showed the activation of distinct gene sets induced by SRS or SRZ. SRZ triggered a pool of defense responses in sorghum, including the upregulation of the genes involved in the regulation of SA biosynthesis, chitin degradation, protein dephosphorylation, ABA signaling and enzymes involved in the modification of the cell wall and membrane lipids. Specifically, enzymes involved in fatty acid and phospholipid degradation, lipid trafficking and sphingolipid biosynthesis, as well as in the metabolism of terpenoids and phenolics were upregulated. This shows that the plant induced defense measures specifically against biotrophic fungi, as well as the generation of phytoalexins. For the *Ustilago maydis*–maize interaction, it was shown that after the induction of an initial defense response at 12 h post-inoculation, the fungus effectively downregulated the defense-induced genes, including the maize SA biosynthesis genes [52]. It could be that when SRZ infects sorghum, it cannot downregulate the plant defense responses as efficiently, or it does not do so any more at three days after inoculation. We did not observe any clear signatures of H_2_O_2_ generation in the transcriptome, likely also because the generation of H_2_O_2_ is observed to happen early (within one or two days) after penetration [5]. Instead, we observed the upregulation of redox mitigators such as thioredoxins and alternative oxidases that work to weaken the destructive effects of H_2_O_2_. Sorghum colonization by SRZ affected several hormone-signaling pathways, including the regulation of SA biosynthesis, the regulation of ABA signaling, as well as the biosynthesis of JA. The functions of ABA are complex [53] and include abiotic stress resistance [54] as well as a negative effect on SA signaling [55]. The upregulation of JA biosynthesis might be induced by the fungus to combat the defensive upregulation of SA biosynthesis by the plant. In any case, it is clear from the transcriptome that the plant recognizes the presence of SRZ and actively tries to combat the intruder.

We unexpectedly observed that the plant responded to the SRZ infection by regulating the genes that putatively lead to a modulation of its lipid content, degrading phospholipids and synthesizing sphingolipids and ceramides, while at the same time inducing lipid trafficking proteins. Sphingolipids have recently been identified to play a critical role in defense against fungal pathogens by directing the initiation of programmed cell death [41]. Possibly, the initiation of programmed cell death in plant cells that are colonized by the fungal hyphae inhibits fungal spread to the meristematic tissues of the sorghum plant [5]. While normally the colonization by *S. reilianum* does not lead to excessive plant cell death [5], the SRZ-induced massive accumulation of the sorghum-specific phytoalexins luteolinidin and apigeninidin can lead to local plant cell death [56]. The inoculation of resistant sorghum with the necrotrophic fungus *B. sorghicola* also led to the induction of plant receptors, MAPK cascades, transcription factors, peroxidases, PR proteins and phytoalexins [28]. Interestingly, the hemibiotroph *Colletotrichum sublineolum* induced defense genes such as chitinases, WRKY transcription factors, MAPK pathway genes and pentatricopeptide repeat genes on both resistant and susceptible cultivars [57]. While in the susceptible cultivar the number of differentially regulated genes was equally high at 48 h as at 24 h post-inoculation, it greatly decreased in the resistant cultivar. This was interpreted to reflect limited fungal growth in the resistant cultivar [57]. In our experiment, we compared the plant transcriptome at three days after inoculation and found a greater number of differentially regulated genes in the inoculation with SRZ. This could indicate that in spite of plant defense reactions, SRZ was still able to actively colonize the leaves and thus lead to the induction of defense genes even at three days after inoculation. Alternatively, a lower number of differentially regulated genes in the SRS-colonized sorghum may reflect a difference in lifestyle, where the suppression of induced plant defenses and/or escaping defense protein recognition may be a success strategy of biotrophic growth behavior.

In contrast to the plant reactions shown upon colonization by SRZ, colonization by SRS did not induce almost any of the defense-related genes, indicating the ability of SRS to successfully evade the host immune system. For example, 21 of the 22 SRZ-induced membrane-spanning kinases were not induced upon colonization by SRS (Table 3). Instead, we observed a strong signature of unfolded protein response in the endoplasmic reticulum lumen of the plant as well as the detoxification of oxidants such as hydrogen peroxide. The unfolded protein response is a general response of the plant towards the stress-induced accumulation of unfolded proteins in the endoplasmic reticulum [58] and has been identified as an important process during plant-microbial immunity [59]. Recently, the endoplasmic reticulum has been shown to respond to and be targeted by bacterial and oomycete effectors [60]. Possibly, SRS also secretes effectors during plant colonization that localize to the endoplasmic reticulum and there induce morphological changes in the process of suppressing plant immunity.

SRS also seems to modify the cuticle composition of the invaded plant. The alkane-forming complex composed of CER1 and CER3 is upregulated, as well as phosphoglycerate kinase that would lead to the generation of the acetyl-CoA needed for the generation of fatty acids and alkanes [61]. In addition, a 3-ketoacyl-CoA synthase and a 3-hydroxy-3-methylglutaryl-CoA synthase are upregulated, that are needed for cuticle wax biosynthesis and plant phytosterol biosynthesis, respectively. Together they could function in modifying the penetrability of the cuticle for SRS or competing microbes.

We did not observe clear signatures of alteration in phytohormone signaling pathways in SRS-inoculated sorghum. This contrasts with other compatible smut–plant interactions. For example, *Sporisorium scitamineum*-inoculated susceptible sugarcane showed an induction of brassinosteroid and auxin signaling at five days after the inoculation of buds and the downregulation of SA, JA and cytokine responses [62]. In contrast, we clearly saw an upregulation of the JA-signaling components in the SRZ–sorghum interaction, which was also observed in *S. scitamineum* on resistant sugarcane cultivars [63]. This may indicate that plant resistance could require the upregulation of JA-signaling components.

The observation that many genes involved in defense are expressed against SRZ is particularly important. This knowledge could be used for the development of strategies to control SRS in sorghum through the generation of plants overexpressing specific plant defenses. Many examples are known where plant defense genes, such as chitinases, β-glucanases and other PR-proteins, were introduced or overexpressed in plants and increased the resistance against pathogens [64,65,66,67].

## 4. Materials and Methods

### 4.1. Plant Lines, Fungal Isolates and Growth Conditions

Plants of *Sorghum bicolor* cv. ‘Tall Polish’ were grown from seed in soil under controlled greenhouse conditions with a 15 h day period at 28 °C and 50% relative humidity, and a 9 h night period at 22 °C and 60% relative humidity. Compatible strains of *Sporisorium reilianum* f. sp. *zeae* SRZ1_5-2 (*a1b1*) and SRZ2_5-1 (*a2b2*), originally isolated from maize [68], and of *Sporisorium reilianum* f. sp. *reilianum* SRS1_H2-8 (*a1b1*) and SRS2_H2-7 (*a2b6*), isolated from sorghum [7] were streaked on a potato dextrose agar (BD, Heidelberg, Germany) and kept at 28 °C for 3–4 days. The strains were then inoculated in 2 mL of YEPS light medium (1% tryptone, 1% yeast extract and 1% sucrose) and were maintained at 28 °C with 200 rpm shaking for about 8 h. The cultures were used to inoculate 50 mL of potato dextrose (2.4%) broth (BD, Heidelberg, Germany) and were kept at 28 °C with shaking until an optical density at 600 nm (OD_600_) of 0.5 to 1.0 was reached. The fungal cultures were pelleted by centrifugation and the cell pellets were suspended in water to reach an OD_600_ of 2.0. A mix of suspensions of SRZ1_5-2 and SRZ2_5-1 (SRZ) or SRS1_H2-8 and SRS2_H2-7 (SRS) were syringe inoculated into the leaf whorls of sorghum seedlings at 14 days after sowing.

### 4.2. RNA Isolation and RNAseq

Sorghum leaves inoculated with SRS, SRZ or water were collected at three days after inoculation. Leaf pieces of about 3 cm (Figure S1) from eight plants were pooled and the experiment was performed for three biological replicates per treatment (Figure S2). The samples were macerated in liquid nitrogen and 100 mg of the resulting powder was collected for RNA extraction with the TRIzol^®^ protocol (Invitrogen, Thermo Fisher Scientific, Darmstadt, Germany). A cleanup step was performed with the RNeasy^®^ Plus Mini Kit (Qiagen, Heidelberg, Germany) and RNA samples were stored at −80 °C. The RNA concentration was determined using a NanoDrop Spectrophotometer (Peqlab, Erlangen, Germany) and RNA integrity was confirmed through denaturing agarose gel electrophoresis. The RNA of the three biological replicates was pooled to reduce sample variation and was sent for Illumina sequencing by an external company (GATC Biotech, Konstanz, Germany).

### 4.3. Read Processing, Mapping and Expression Analysis

The Tuxedo protocol [32], a set of LINUX-based command line tools, was used for the analysis of the reads, including the trimming of adapters and differential gene expression analysis. The adapters were trimmed using Trimmomatic 0.36; trimmed reads were assembled using TopHat 2.1.0 and were mapped to the genome of *S. bicolor* GCA_000003195.3_Sorghum_bicolor_NCBIv3 [21,33] using Cufflinks 2.2.1 [32]. The read counts were normalized with FPKM and assemblies were merged using Cuffmerge 2.2.1. Cuffdiff 2.2.1 was used to compute the differentially expressed genes between the three conditions (SRS vs. control, SRZ vs. control, SRS vs. SRZ). The resulting file was rearranged to show each sorghum gene locus in only one line and all lines lacking a SORBI_ID in the “gene” column were manually deleted (Table S1). Some loci (917) corresponded to more than one SORBI_ID; these were treated as one gene. Log2 fold change expression values were calculated after adding a constant (0.000001) to each value to avoid zero scores during log calculations.

### 4.4. Analyzing Differential Gene Expression Using MapMan and GO Enrichment

The genes significantly up- and downregulated in the comparison of the SRS-infected plants versus the water control, the SRZ-infected plants versus the water control and the SRZ- versus SRS-infected plants were selected for annotation using the MapMan program (version 3.5.1R2, https://mapman.gabipd.org/home, Forschungszentrum Jülich, Jülich, Germany) [34] and were used for GO enrichment analysis by the Gene Ontology Resource [69].

### 4.5. Gene Expression Validation by Real-Time PCR

Eight sorghum genes that showed differential expression among the three treatments (SRS, SRZ and water control) were selected for validation via qRT-PCR. For that, leaf whorls of 14-day-old sorghum seedlings were syringe inoculated with suspensions of SRZ1_5-2 and SRZ2_5-1 (SRZ) or SRS1_H2-8 and SRS2_H2-7 (SRS). The inoculation was performed in three biological replicates of 17 plants each. The leaf samples were collected three days after inoculation, as previously described. Samples of each replicate were pooled and RNA was isolated using ROTI^®^Aqua-Phenol following the TRIzol^®^ protocol. The following DNAse treatment using Invitrogen™ DNaseI (Thermo Fisher Scientific, Darmstadt, Germany) and subsequent first strand cDNA synthesis using Thermo Scientific™ RevertAid Reverse Transcriptase (Thermo Fisher Scientific, Darmstadt, Germany) were performed according to the manufacturer’s instructions. The qPCR-Primers used are listed in Table S2. Primer efficiencies were tested and were between 85 and 100%. Elongation factor 4 alpha (*ElF4α*, SORBI_3004G039400) and protein phosphatase 2 (*PP2A*, SORBI_3004G092500) were selected as reference genes [70]. A real-time PCR was performed in a CFX 96 Thermal Cycler (Bio-Rad Laboratories, Hercules, CA, USA) in a 10 µL reaction mixture using Luna^®^ Universal qPCR Master Mix (NEB, Germany) and 20 ng of a cDNA template. The PCR conditions consisted of an initial denaturation at 95 °C for 1 min followed by 40 cycles of 95 °C for 30 s, 58 °C for 30 s, 60 °C for 30 s, a plate read step and lastly a product melting curve at 60–95 °C. Three technical replicates were performed for each biological replicate. Expression ratios in the samples of the inoculated plants were calculated using the CFX Manager 3.0 (Bio-Rad Laboratories, Hercules, CA, USA).

### 4.6. Accession Numbers

The transcriptome data were deposited at the Genomic Expression Archive (GEA) under the BioProject ID PRJNA750474 with the BioSample accessions SAMN20460444, SAMN20460445, SAMN20460446.

## 5. Conclusions

In this paper, we present a comparative transcriptome analysis of *Sorghum bicolor* inoculated with the two *formae speciales* SRS and SRZ of *S. reilianum*, which can both colonize leaves but only the former is able to cause the typical smut symptoms. We showed that both SRS and SRZ lead to gene expression rather than suppression and that both trigger very different plant gene expression responses in sorghum. The non-virulent *forma specialis* SRZ induces a multitude of defense responses in sorghum that are mainly associated with the plasma membrane, including the exchange of phospholipids by sphingolipids, and the generation of defense compounds such as terpenes and phytoalexins. In contrast, SRS induces only relatively few genes that are involved in the control of oxidative damage and protein folding, mainly in the ER. This identifies the endoplasmic reticulum of the plant as a putatively important compartment that may allow plant susceptibility towards the pathogen, while the lipid composition of the membrane may be involved in resistance. This may open a novel line of future research with increased attention on the contribution of the plant endoplasmic reticulum and the plant plasma membrane on infection success by fungal pathogens. To better understand the differential response of the plant, a time course of gene expression changes in sorghum during colonization by the two *formae speciales* of *S. reilianum* in different sorghum tissues would be desirable.

## Figures and Tables

**Table 1:** Overview of mapped reads.

Sample	Sb-SRS		Sb-SRZ		Sb-H_2_O	
	Number of Reads (%)		Number of Reads (%)		Number of Reads (%)	
All	44.513.277	(100.0) ^a^	46.807.963	(100.0) ^a^	88.930.659	(100.0) ^a^
Mapped	42.365.430	(95.2) ^a^	43.347.104	(92.6) ^a^	85.460.918	(96.1) ^a^
Non-unique	2.857.609	(6.7) ^b^	1.953.455	(4.5) ^b^	4.306.028	(5.0) ^b^
Mapped > 20×	6.871	(0.2) ^c^	7.876	(0.4) ^c^	17.373	(0.4) ^c^
Unique	39.507.871	(93.3) ^b^	41.393.649	(95.5) ^b^	81.154.890	(95.0) ^b^

^a^ % of all reads, ^b^ % of all mapped reads, ^c^ % of all non-unique mapped reads.

**Table 2:** Metabolic plant genes depicted in Figure 6 as significantly upregulated in either SRS- or SRZ-inoculated sorghum.

			Log2 (Fold Change) in the Comparison	
Transcript ID	Gene ID	Possible Function	Sb-SRZ vs. Sb-H_2_O	Sb-SRS vs. Sb-H_2_O
eer97763	SORBI_3002G423600	Galactinol synthase	3.83	
oqu88584	SORBI_3002G057900	H-type thioredoxin	2.78	
eer94707	SORBI_3001G342600	Nucleoredoxin	2.47	
kxg30341	SORBI_3004G166700	Acid beta-fructofuranosidase (CWIN)	3.67	4.02
oqu75996	SORBI_3010G072300	Sucrose synthase	2.56	
kxg36452	SORBI_3002G334500	D-glucan synthase (CSLF)	3.65	
kxg33870	SORBI_3003G384700	Pectin acetylesterase	3.33	
ees17845	SORBI_3009G071800	ATP-dependent phosphofructokinase	4.35	
kxg33170	SORBI_3003G265100	Phosphatidate phosphatase (LPP-alpha)	3.96	
ees14083	SORBI_3007G168000	Chlorophyllase (CLH)	2.81	
eer92546	SORBI_3001G434900	Catalytic subunit 1 of serine C-palmitoyltransferase complex	3.56	
eer98437	SORBI_3002G122700	Small regulatory subunit of serine C-palmitoyltransferase complex	3.5	
ees06132	SORBI_3004G008300	Sphingobase hydroxylase	16.14	
kxg23697	SORBI_3008G129000	Inositol phosphorylceramide synthase (IPCS)	3.74	
oqu92667	SORBI_3001G386400	Active component ALA of ALA-ALIS flippase complex	3.9	
ees17516	SORBI_3009G000200	Active component ALA of ALA-ALIS flippase complex	4.74	
ees14950	SORBI_3007G119800	Fatty acid transporter (ABCA)	3.14	
oqu87824	SORBI_3003G348700	Monoacylglycerol lipase	6.91	
eer89541	SORBI_3010G104600	Caleosin	5.88	
ees18897	SORBI_3009G014600	Phospholipase A1 (PC-PLA1)	7.28	
oqu88219	SORBI_3003G432600	Phospholipase A1 (PC-PLA1)	6.2	
ees10106	SORBI_3005G186100	Phospholipase A2 (pPLA2-II)	5.8	
ees08840	SORBI_3005G186200	Phospholipase A2 (pPLA2-II)	4.11	
kxg39221	SORBI_3001G349800	Phospholipase D (PLD-alpha)	4.83	
ees10287	SORBI_3005G222500	Phospholipase D (PLD-alpha)	15.79	
eer94131	SORBI_3001G230100	3-Ketoacyl-CoA thiolase (KAT)	3.27	
ees14522	SORBI_3007G035700	NAD(P)H dehydrogenase (NDB)	2.9	
ees12783	SORBI_3006G203000	Alternative oxidase (Aox)	5.82	
ees12781	SORBI_3006G202500	Alternative oxidase (Aox)	3.68	
ees10479	SORBI_3006G026900	Phosphate transporter (PHT1)	4.28	
kxg34441	SORBI_3002G041200	Nicotianamine amino transferase	4.8	
ees04390	SORBI_3004G018900	Mono-/sesquiterpene-/diterpene synthase	18.06	
ees04394	SORBI_3004G019300	Mono-/sesquiterpene-/diterpene synthase	5.11	
ees04392	SORBI_3004G019100	Mono-/sesquiterpene-/diterpene synthase	5.31	
oqu81208	SORBI_3006G027500	Mono-/sesquiterpene-/diterpene synthase	4.28	
kxg24531	SORBI_3007G055600	Mono-/sesquiterpene-/diterpene synthase	7.79	
kxg24530	SORBI_3007G055500	Mono-/sesquiterpene-/diterpene synthase	8.79	
eer94760	SORBI_3001G351000	3-Deoxy-D-arabino-heptulosonate 7-phosphate (DAHP) synthase	2.98	2.89
ees03803	SORBI_3003G337400	Cinnamate 4-hydroxylase (C4H)	3.59	
kxg29016	SORBI_3005G136800	Chalcone synthase	18.34	
ees09862	SORBI_3005G137000	Chalcone synthase	7.98	
ees09858	SORBI_3005G136300	Chalcone synthase	6.1	
ees09863	SORBI_3005G137100	Chalcone synthase	7.5	
eer92960	SORBI_3001G526900	Type-I flavone synthase	5.66	
eer94585	SORBI_3001G314300	Type-I flavone synthase	6.01	
eer95206	SORBI_3001G443800	Glutamate decarboxylase	3.65	
kxg20563	SORBI_3010G221800	Phosphoglycerate kinase		3.66
oqu93373	SORBI_3001G530100	3-Hydroxy-3-methylglutaryl-CoA synthase		3.21
kxg30650	SORBI_3004G218100	Aldehyde decarbonylase component CER1		3.3
eer88750	SORBI_3010G212600	Aldehyde-generating component CER3		4.39
eer95458	SORBI_3001G495500	3-Ketoacyl-CoA synthase (KCS)	5.31	

**Table 3:** Sorghum kinases upregulated in the comparison of Sb-SRZ vs. Sb-H_2_O or Sb-SRS vs. Sb-H_2_O.

Transcript Identifier	Protein Family	Log2 FC Sb-SRZ vs. Sb-H_2_O	Log2 FC Sb-SRS vs. Sb-H_2_O
*Membrane-spanning kinases*			
Eer97932	L-type lectin	4.4	n.d. ^1^
Ees03486	MAP3K/MEKK	14.7	n.d.
Oqu85116		4.1	n.d.
Oqu80963	WAK-like	4.6	n.d.
Oqu81999		14.5	n.d.
Oqu88391		5.2	n.d.
Kxg36444	S-domain	5.5	n.d.
Eer99485		4.6	n.d.
Eer99486		3.9	n.d.
Oqu82405		3.9	n.d.
Kxg39626	CMGC	2.5	n.d.
Kxg33373	PERK	3.2	n.d.
Eer99485	DUF26	4.6	n.d.
Eer99486		3.9	n.d.
Eer97562	SNF-1 related (SnRK2)	3.1	n.d.
Oqu88438		3.5	n.d.
Oqu87587		14.8	n.d.
Ees03800	LRR-VIII-1	3.3	n.d.
Kxg23128	LRR-Xc	3.2	n.d.
Kxg25252	LRR-XI	4.0	n.d.
Ees12871	LRR-XII	4.3	5.5
Kxg20259		4.5	n.d.
*Cytoplasmic kinases*			
Eer90813	LRR-XIV	3.0	n.d.
Oqu87509	LRR-XV	5.0	n.d.
Ees01457		6.4	n.d.
Ees06537		4.4	n.d.
Ees03807	RLCK-II	4.2	n.d.
Eer99248	RLCK-VIIa	3.3	n.d.
Kxg19254	RLCK-IXb	3.3	n.d.
Oqu83247	DLSV	4.0	n.d.
Kxg28202		4.0	n.d.

^1^ n.d., not detected in the dataset as regulated.

## Data Availability

Not applicable.

## Supplementary Materials

The supporting information can be downloaded at: https://www.mdpi.com/article/10.3390/ijms23168864/s1.

## References

[1] Hossain S., Islam N., Rahman M., Mostofa M.G., Khan A.R. (2022). Sorghum: A prospective crop for climatic vulnerability, food and nutritional security. J. Agri. Food Res..

[2] Halisky P. (1963). Head smut of sorghum, sudangrass, and corn, caused by *Sphacelotheca reiliana* (Kühn) Clint. Hilgardia.

[3] Matyac C.A., Kommedahl T. (1986). Survival of teliospores of *Sphacelotheca reiliana* in soil. Phytopathology.

[4] Schirawski J., Heinze B., Wagenknecht M., Kahmann R. (2005). Mating type loci of *Sporisorium reilianum*: Novel pattern with three *a* and multiple *b* specificities. Eukaryot. Cell.

[5] Poloni A., Schirawski J. (2016). Host specificity in *Sporisorium reilianum* is determined by distinct mechanisms in maize and sorghum. Mol. Plant Pathol..

[6] Sohaily A.L., Mankin H.J. (1960). Reaction of corn and sorghum to corn and sudan grass head smuts. Plant Dis. Rep..

[7] Zuther K., Kahnt J., Utermark J., Imkampe J., Uhse S., Schirawski J. (2012). Host specificity of *Sporisorium reilianum* is tightly linked to generation of the phytoalexin luteolinidin by *Sorghum bicolor*. Mol. Plant Microbe Interact..

[8] Ahuja I., Kissen R., Bones A.M. (2012). Phytoalexins in defense against pathogens. Trends Plant Sci..

[9] Chinchilla D., Bauer Z., Regenass M., Boller T., Felix G. (2006). The Arabidopsis receptor kinase FLS2 binds flg22 and determines the specificity of flagellin perception. Plant Cell.

[10] Miya A., Albert P., Shinya T., Desaki Y., Ichimura K., Shirasu K., Narusaka Y., Kawakami N., Kaku H., Shibuya N. (2007). CERK1, a LysM receptor kinase, is essential for chitin elicitor signaling in Arabidopsis. Proc. Natl. Acad. Sci. USA.

[11] Monaghan J., Zipfel C. (2012). Plant pattern recognition receptor complexes at the plasma membrane. Curr. Opin. Plant Biol..

[12] Underwood W. (2012). The plant cell wall: A dynamic barrier against pathogen invasion. Front. Plant Sci..

[13] Van Loon L.C., Rep M., Pieterse C.M. (2006). Significance of inducible defense-related proteins in infected plants. Annu. Rev. Phytopathol..

[14] Zipfel C., Robatzek S., Navarro L., Oakeley E.J., Jones J.D., Felix G., Boller T. (2004). Bacterial disease resistance in Arabidopsis through flagellin perception. Nature.

[15] Pasini L., Bergonti M., Fracasso A., Marocco A., Amaducci S. (2014). Microarray analysis of differentially expressed mRNAs and miRNAs in young leaves of sorghum under dry-down conditions. J. Plant Physiol..

[16] Buchanan C.D., Lim S., Salzman R.A., Kagiampakis I., Morishige D.T., Weers B.D., Klein R.R., Pratt L.H., Cordonnier-Pratt M.M., Klein P.E. (2005). *Sorghum bicolor*’s transcriptome response to dehydration, high salinity and ABA. Plant Mol. Biol..

[17] Salzman R.A., Brady J.A., Finlayson S.A., Buchanan C.D., Summer E.J., Sun F., Klein P.E., Klein R.R., Pratt L.H., Cordonnier-Pratt M.M. (2005). Transcriptional profiling of sorghum induced by methyl jasmonate, salicylic acid, and aminocyclopropane carboxylic acid reveals cooperative regulation and novel gene responses. Plant Physiol..

[18] Park S.J., Huang Y., Ayoubi P. (2006). Identification of expression profiles of sorghum genes in response to greenbug phloem-feeding using cDNA subtraction and microarray analysis. Planta.

[19] Zhu-Salzman K., Salzman R.A., Ahn J.E., Koiwa H. (2004). Transcriptional regulation of sorghum defense determinants against a phloem-feeding aphid. Plant Physiol..

[20] Cooper E.A., Brenton Z.W., Flinn B.S., Jenkins J., Shu S., Flowers D., Luo F., Wang Y., Xia P., Barry K. (2019). A new reference genome for *Sorghum bicolor* reveals high levels of sequence similarity between sweet and grain genotypes: Implications for the genetics of sugar metabolism. BMC Genom..

[21] Paterson A.H., Bowers J.E., Bruggmann R., Dubchak I., Grimwood J., Gundlach H., Haberer G., Hellsten U., Mitros T., Poliakov A. (2009). The *Sorghum bicolor* genome and the diversification of grasses. Nature.

[22] Dugas D.V., Monaco M.K., Olsen A., Klein R.R., Kumari S., Ware D., Klein P.E. (2011). Functional annotation of the transcriptome of *Sorghum bicolor* in response to osmotic stress and abscisic acid. BMC Genom..

[23] Chopra R., Burow G., Hayes C., Emendack Y., Xin Z.G., Burke J. (2015). Transcriptome profiling and validation of gene based single nucleotide polymorphisms (SNPs) in sorghum genotypes with contrasting responses to cold stress. BMC Genom..

[24] Gelli M., Duo Y., Konda A.R., Zhang C., Holding D., Dweikat I. (2014). Identification of differentially expressed genes between sorghum genotypes with contrasting nitrogen stress tolerance by genome-wide transcriptional profiling. BMC Genom..

[25] Doring F., Streubel M., Brautigam A., Gowik U. (2016). Most photorespiratory genes are preferentially expressed in the bundle sheath cells of the C4 grass *Sorghum bicolor*. J. Exp. Bot..

[26] Wu X.Y., Hu W.J., Luo H., Xia Y., Zhao Y., Wang L.D., Zhang L.M., Luo J.C., Jing H.C. (2016). Transcriptome profiling of developmental leaf senescence in sorghum (*Sorghum bicolor*). Plant Mol. Biol..

[27] Mizuno H., Kawahigashi H., Kawahara Y., Kanamori H., Ogata J., Minami H., Itoh T., Matsumoto T. (2012). Global transcriptome analysis reveals distinct expression among duplicated genes during sorghum-*Bipolaris sorghicola* interaction. BMC Plant Biol..

[28] Yazawa T., Kawahigashi H., Matsumoto T., Mizuno H. (2013). Simultaneous transcriptome analysis of sorghum and *Bipolaris sorghicola* by using RNA-seq in combination with de novo transcriptome assembly. PLoS ONE.

[29] Abdel-Ghany S.E., Hamilton M., Jacobi J.L., Ngam P., Devitt N., Schilkey F., Ben-Hur A., Reddy A.S.N. (2016). A survey of the sorghum transcriptome using single-molecule long reads. Nat. Commun..

[30] Varoquaux N., Cole B., Gao C., Pierroz G., Baker C.R., Patel D., Madera M., Jeffers T., Hollingsworth J., Sievert J. (2019). Transcriptomic analysis of field-droughted sorghum from seedling to maturity reveals biotic and metabolic responses. Proc. Natl. Acad. Sci. USA.

[31] Zhang J., Jiang F., Shen Y., Zhan Q., Bai B., Chen W., Chi Y. (2019). Transcriptome analysis reveals candidate genes related to phosphorus starvation tolerance in sorghum. BMC Plant Biol..

[32] Trapnell C., Roberts A., Goff L., Pertea G., Kim D., Kelley D.R., Pimentel H., Salzberg S.L., Rinn J.L., Pachter L. (2012). Differential gene and transcript expression analysis of RNA-seq experiments with TopHat and Cufflinks. Nat. Protoc..

[33] McCormick R.F., Truong S.K., Sreedasyam A., Jenkins J., Shu S., Sims D., Kennedy M., Amirebrahimi M., Weers B.D., McKinley B. (2018). The *Sorghum bicolor* reference genome: Improved assembly, gene annotations, a transcriptome atlas, and signatures of genome organization. Plant J..

[34] Thimm O., Blasing O., Gibon Y., Nagel A., Meyer S., Kruger P., Selbig J., Muller L.A., Rhee S.Y., Stitt M. (2004). MAPMAN: A user-driven tool to display genomics data sets onto diagrams of metabolic pathways and other biological processes. Plant J..

[35] Jones J.D., Henstrand J.M., Handa A.K., Herrmann K.M., Weller S.C. (1995). Impaired wound induction of 3-deoxy-D-arabino-heptulosonate-7-phosphate (DAHP) synthase and altered stem development in transgenic potato plants expressing a DAHP synthase antisense construct. Plant Physiol..

[36] Proels R.K., Hückelhoven R. (2014). Cell-wall invertases, key enzymes in the modulation of plant metabolism during defence responses. Mol. Plant Pathol..

[37] Liao P., Wang H., Hemmerlin A., Nagegowda D.A., Bach T.J., Wang M., Chye M.L. (2014). Past achievements, current status and future perspectives of studies on 3-hydroxy-3-methylglutaryl-CoA synthase (HMGS) in the mevalonate (MVA) pathway. Plant Cell Rep..

[38] Bourdenx B., Bernard A., Domergue F., Pascal S., Leger A., Roby D., Pervent M., Vile D., Haslam R.P., Napier J.A. (2011). Overexpression of Arabidopsis *ECERIFERUM1* promotes wax very-long-chain alkane biosynthesis and influences plant response to biotic and abiotic stresses. Plant Physiol..

[39] Chen M., Han G.S., Dietrich C.R., Dunn T.M., Cahoon E.B. (2006). The essential nature of sphingolipids in plants as revealed by the functional identification and characterization of the Arabidopsis LCB1 subunit of serine palmitoyltransferase. Plant Cell.

[40] Gault C.R., Obeid L.M., Hannun Y.A. (2010). An overview of sphingolipid metabolism: From synthesis to breakdown. Adv. Exp. Med. Biol..

[41] Berkey R., Bendigeri D., Xiao S.Y. (2012). Sphingolipids and plant defense/disease: The “death” connection and beyond. Front. Plant Sci..

[42] Poloni A., Schirawski J. (2014). Red card for pathogens: Phytoalexins in sorghum and maize. Molecules.

[43] Dao T.T., Linthorst H.J., Verpoorte R. (2011). Chalcone synthase and its functions in plant resistance. Phytochem. Rev..

[44] Falcone Ferreyra M.L., Emiliani J., Rodriguez E.J., Campos-Bermudez V.A., Grotewold E., Casati P. (2015). The identification of maize and Arabidopsis type I FLAVONE SYNTHASEs links flavones with hormones and biotic interactions. Plant Physiol..

[45] Nishizawa A., Yabuta Y., Shigeoka S. (2008). Galactinol and raffinose constitute a novel function to protect plants from oxidative damage. Plant Physiol..

[46] Walder F., Brule D., Koegel S., Wiemken A., Boller T., Courty P.E. (2015). Plant phosphorus acquisition in a common mycorrhizal network: Regulation of phosphate transporter genes of the Pht1 family in sorghum and flax. New Phytol..

[47] Seifi H.S., Van Bockhaven J., Angenon G., Hofte M. (2013). Glutamate metabolism in plant disease and defense: Friend or foe?. Mol. Plant Microbe Interact..

[48] Kariola T., Brader G., Li J., Palva E.T. (2005). Chlorophyllase 1, a damage control enzyme, affects the balance between defense pathways in plants. Plant Cell.

[49] Higuchi K., Suzuki K., Nakanishi H., Yamaguchi H., Nishizawa N.K., Mori S. (1999). Cloning of nicotianamine synthase genes, novel genes involved in the biosynthesis of phytosiderophores. Plant Physiol..

[50] Greenshields D.L., Liu G., Wei Y. (2007). Roles of iron in plant defence and fungal virulence. Plant Signal. Behav..

[51] Suleman M., Ma M., Ge G., Hua D., Li H. (2021). The role of alternative oxidase in plant hypersensitive response. Plant Biol..

[52] Doehlemann G., Wahl R., Horst R.J., Voll L.M., Usadel B., Poree F., Stitt M., Pons-Kuhnemann J., Sonnewald U., Kahmann R. (2008). Reprogramming a maize plant: Transcriptional and metabolic changes induced by the fungal biotroph *Ustilago maydis*. Plant J..

[53] Lievens L., Pollier J., Goossens A., Beyaert R., Staal J. (2017). Abscisic acid as pathogen effector and immune regulator. Front. Plant Sci..

[54] Sah S.K., Reddy K.R., Li J. (2016). Abscisic acid and abiotic stress tolerance in crop plants. Front. Plant Sci..

[55] Jiang C.J., Shimono M., Sugano S., Kojima M., Yazawa K., Yoshida R., Inoue H., Hayashi N., Sakakibara H., Takatsuji H. (2010). Abscisic acid interacts antagonistically with salicylic acid signaling pathway in rice-*Magnaporthe grisea* interaction. Mol. Plant Microbe Interact..

[56] Nielsen K.A., Gotfredsen C.H., Buch-Pedersen M.J., Ammitzbøll H., Mattsson O., Duus J.Ø., Nicholson R.L. (2004). Inclusions of flavonoid 3-deoxyanthocyanidins in *Sorghum bicolor* self-organize into spherical structures. Physiol. Mol. Plant Pathol..

[57] Fu F., Girma G., Mengiste T. (2020). Global mRNA and microRNA expression dynamics in response to anthracnose infection in sorghum. BMC Genom..

[58] Hartl F.U., Hayer-Hartl M. (2009). Converging concepts of protein folding in vitro and in vivo. Nat. Struct. Mol. Biol..

[59] Bao Y., Howell S.H. (2017). The unfolded protein response supports plant development and defense as well as responses to abiotic stress. Front. Plant Sci..

[60] Breeze E., Vale V., McLellan H., Godiard L., Grant M., Frigerio L. (2020). The plant endoplasmic reticulum is both receptive and responsive to pathogen effectors. bioRxiv.

[61] Bernard A., Domergue F., Pascal S., Jetter R., Renne C., Faure J.-D., Haslam R.P., Napier J.A., Lessire R., Joubès J. (2012). Reconstitution of plant alkane biosynthesis in yeast demonstrates that Arabidopsis ECERIFERUM1 and ECERIFERUM3 are core components of a very-long-chain alkane synthesis complex. Plant Cell.

[62] Schaker P.D., Palhares A.C., Taniguti L.M., Peters L.P., Creste S., Aitken K.S., Van Sluys M.A., Kitajima J.P., Vieira M.L., Monteiro-Vitorello C.B. (2016). RNAseq transcriptional profiling following whip development in sugarcane smut disease. PLoS ONE.

[63] Que Y., Su Y., Guo J., Wu Q., Xu L. (2014). A global view of transcriptome dynamics during *Sporisorium scitamineum* challenge in sugarcane by RNA-Seq. PLoS ONE.

[64] Gomez-Ariza J., Campo S., Rufat M., Estopa M., Messeguer J., San Segundo B., Coca M. (2007). Sucrose-mediated priming of plant defense responses and broad-spectrum disease resistance by overexpression of the maize pathogenesis-related PRms protein in rice plants. Mol. Plant Microbe Interact..

[65] Iwai T., Kaku H., Honkura R., Nakamura S., Ochiai H., Sasaki T., Ohashi Y. (2002). Enhanced resistance to seed-transmitted bacterial diseases in transgenic rice plants overproducing an oat cell-wall-bound thionin. Mol. Plant Microbe Interact..

[66] Jach G., Gornhardt B., Mundy J., Logemann J., Pinsdorf E., Leah R., Schell J., Maas C. (1995). Enhanced quantitative resistance against fungal disease by combinatorial expression of different barley antifungal proteins in transgenic tobacco. Plant J..

[67] Zhu Q., Maher E.A., Masoud S., Dixon R.A., Lamb C.J. (1994). Enhanced protection against fungal attack by constitutive co–expression of chitinase and glucanase genes in transgenic tobacco. Nat. Biotechnol..

[68] Schirawski J., Mannhaupt G., Munch K., Brefort T., Schipper K., Doehlemann G., Di Stasio M., Rossel N., Mendoza-Mendoza A., Pester D. (2010). Pathogenicity determinants in smut fungi revealed by genome comparison. Science.

[69] Mi H., Muruganujan A., Ebert D., Huang X., Thomas P.D. (2019). PANTHER version 14: More genomes, a new PANTHER GO-slim and improvements in enrichment analysis tools. Nucleic Acids Res..

[70] Reddy D.S., Bhatnagar-Mathur P., Reddy P.S., Sri Cindhuri K., Sivaji Ganesh A., Sharma K.K. (2016). Identification and validation of reference genes and their impact on normalized gene expression studies across cultivated and wild *Cicer* species. PLoS ONE.

