# Supplementary figures and images for: Transcriptome Analysis Reveals Contrasting Plant Responses of Sorghum bicolor upon Colonization by Two Formae Speciales of Sporisorium reilianum
Source: Int J Mol Sci. 2022 Aug 9;23(16):8864. doi: 10.3390/ijms23168864 (PMC9407964; doi:10.3390/ijms23168864)

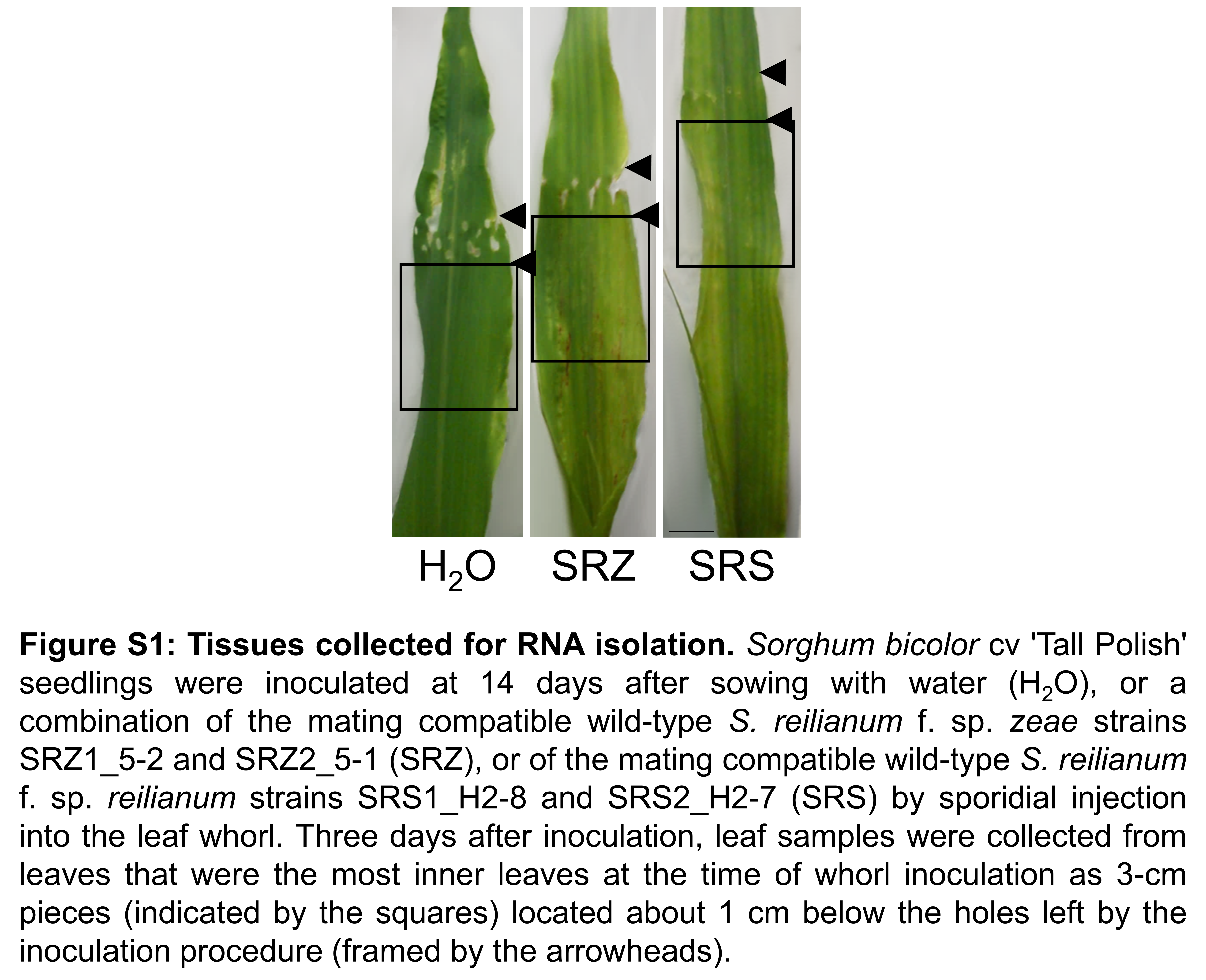

Supplement: Supplementary file 1 [file ijms-23-08864-s001.zip › Figure S1 for export.tif]
